# 3M_BANTOR: A regression framework for multitask and multisession brain network distance metrics

**DOI:** 10.1162/netn_a_00274

**Published:** 2023-01-01

**Authors:** Chal E. Tomlinson, Paul J. Laurienti, Robert G. Lyday, Sean L. Simpson

**Affiliations:** Department of Biostatistics, University of North Carolina at Chapel Hill, Chapel Hill, NC, USA; Laboratory for Complex Brain Networks, Wake Forest University School of Medicine, Winston-Salem, NC, USA; Department of Radiology, Wake Forest University School of Medicine, Winston-Salem, NC, USA; Department of Biostatistics and Data Science, Wake Forest University School of Medicine, Winston-Salem, NC, USA

**Keywords:** Graph theory, Connectivity, fMRI, Neuroimaging, Jaccard, Kolmogorov–Smirnov, Log-Euclidean Riemannian metric, Riemannian manifold distance, Mixed model, Repeated observations, Human Connectome Project (HCP), Pearson correlation distance

## Abstract

Brain network analyses have exploded in recent years and hold great potential in helping us understand normal and abnormal brain function. Network science approaches have facilitated these analyses and our understanding of how the brain is structurally and functionally organized. However, the development of statistical methods that allow relating this organization to phenotypic traits has lagged behind. Our previous work developed a novel analytic framework to assess the relationship between brain network architecture and phenotypic differences while controlling for confounding variables. More specifically, this innovative regression framework related distances (or similarities) between brain network features from a single task to functions of absolute differences in continuous covariates and indicators of difference for categorical variables. Here we extend that work to the multitask and multisession context to allow for multiple brain networks per individual. We explore several similarity metrics for comparing distances between connection matrices and adapt several standard methods for estimation and inference within our framework: standard *F* test, *F* test with scan-level effects (SLE), and our proposed mixed model for multitask (and multisession) BrAin NeTwOrk Regression (3M_BANTOR). A novel strategy is implemented to simulate symmetric positive-definite (SPD) connection matrices, allowing for the testing of metrics on the Riemannian manifold. Via simulation studies, we assess all approaches for estimation and inference while comparing them with existing multivariate distance matrix regression (MDMR) methods. We then illustrate the utility of our framework by analyzing the relationship between fluid intelligence and brain network distances in Human Connectome Project (HCP) data.

## INTRODUCTION

As brain network analyses have exploded in recent years, neuroimaging researchers often face the need to statistically compare brain networks (Simpson et al., 2013a). Many approaches for relating brain networks to clinical outcomes or demographical variables have been developed. Such methods include but are not limited to traditional network models (e.g., exponential random graph models; Lehmann et al., 2021; Simpson et al., 2011, 2012), tensor regression works on brain network (e.g., Zhang et al., 2018, 2019), Bayesian approaches (e.g., Dai & Guo, 2017; Wang et al., 2017), statistical learning techniques (Craddock et al., 2015; Varoquaux & Craddock, 2013; Xia et al., 2020), and testing based on distance correlation (Székely et al., 2007; Székely & Rizzo, 2009). Despite the advances made, analysis methods are still needed that enable relating brain network organization to phenotypic traits. In order to develop such an analysis, we can exploit the fact that brain networks often exhibit consistent organizations across subjects. Toward this end, in previous work we developed a permutation testing framework that detects whether the spatial location of network features (such as the location of high degree nodes) mapped back into brain space differs between two groups of networks, and whether distributions of topological properties vary by group (Simpson et al., 2013b). We then proposed an innovative regression framework to relate distances between brain network features from a single task to functions of absolute differences in continuous covariates and indicators of difference for categorical variables (Tomlinson et al., 2022). Here we extend that work to the multitask and multisession context to allow for multiple brain networks per individual.

We considered several different types of metrics for establishing distances (i.e., similarity/dissimilarity) between networks. The first type compares degree distributions. We accomplish this by summarizing similarities in connection-based degree distributions across multiple networks with the Kolmogorov–Smirnov statistic (KS statistic), a measure that quantifies the distance between two cumulative distribution functions (Kolmogorov, 1933; Smirnov, 1948). The second type takes into account consistency of key edge sets. We do so by summarizing similarities in edge sets across multiple networks with the Jaccard distance (or Jaccard index), a metric that quantifies difference (or similarity) in partitions of a set (Joyce et al., 2010; Meunier et al., 2009). The third type of metric measures differences in network edges by employing the Euclidean distance between connectivity matrices (Lance & Williams, 1966). The fourth type of metric (similar to the third) measures correlations in network edges by employing the Pearson correlation distance between connectivity matrices (van Dongen & Enright, 2012). The fourth metric is also edge-based but measures differences between connectivity matrices with the log-Euclidean Riemannian metric (Arsigny et al., 2006). The log-Euclidean Riemannian metric (LERM) is a metric not considered in our previous work and is used as a computationally friendly approximation of the affine-invariant Riemannian metric (AIRM). Riemannian metrics are used to measure representational connectivity (Shahbazi et al., 2021) and require the use of symmetric positive-definite (SPD) matrices.

While our previous work summed over the rows of connection matrices to show the utility of comparing nodal degree vectors, this work focuses solely on distance metrics utility by using entire connection matrices. This changes the interpretation of what a difference means, that is, switching the individual comparisons from nodal degrees to edge weights. However, all metrics discussed here, except for LERM, are able to handle nodal degree vectors as well. There is evidence of edge-centric functional connectivity exhibiting consistent organizations across subjects over multiple scan sessions (Finn et al., 2015; Shen et al., 2017).

Within our regression framework we adapt several methods for estimation and inference: standard *F* test, *F* test with SLE, and our proposed mixed model for multitask (and multisession) BrAin NeTwOrk Regression (3M_BANTOR). Each observation in the regression framework includes a “distance” between two individuals, so observations that share individuals are correlated. Thus, the standard *F* test is generally not appropriate but presented for comparison. Since distances between individuals will be repeated, as individuals have multiple scans each, we should not expect including fixed SLE within the regression to render the *F* test valid. It is presented here for comparison as well, as this was our chosen method when each individual had only one scan (Tomlinson et al., 2022). 3M_BANTOR includes scan-level fixed effects as well as random effects to account for repetitions among individuals in an attempt to handle the correlation induced by including multiple scans per individual.

As mentioned previously, many existing methods exist for relating network metrics and phenotypes. We believe our method most closely relates to multivariate distance matrix regression (MDMR). MDMR tests the significance of associations of response profile (dis)similarities and a set of predictors. Originally this was done using only permutation tests (Anderson, 2001), but later extended to analytic *p* values and nonindependent observations (McArtor, 2017). These MDMR methods will be considered for comparison with our proposed methods (*F* test, *F* test with SLE, and 3M_BANTOR).

In this paper, we detail our regression framework and discuss several methods for estimation and inference to be used with a variety of network similarity/dissimilarity metrics within the framework. A novel strategy is implemented to simulate SPD connection matrices, allowing for the testing of metrics on the Riemannian manifold. We assess all combinations of methods and metrics within this framework by using simulated fMRI data with known differences in connectivity matrix distributions. We then apply our framework to multitask and multisession functional brain networks derived from the HCP dataset to investigate the relationship between fluid intelligence and network distances after accounting for known confounders.

## METHODS

Please note the following notational choices: bold font is used to denote vectors or matrices, *n* = number of observations, *n*_*p*_ = number of participants, *n*_*n*_ = number of nodes, *n*_*t*_ = number of tasks, *n*_*r*_ = number of repetitions (of a given task), *p* = number of covariates (including intercept, if included).

### Step 1: Network Construction

**Figure 1.**
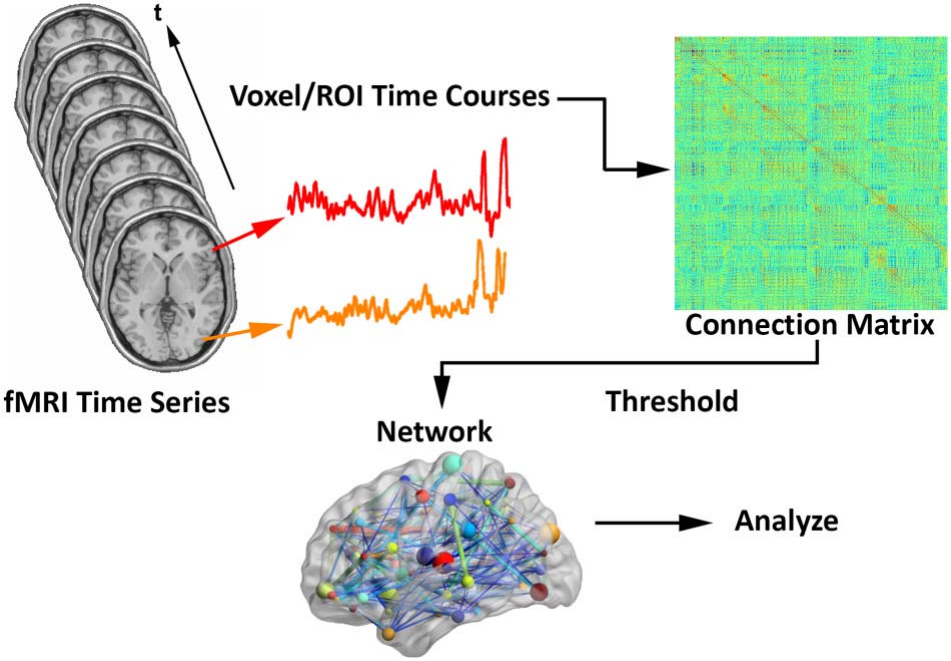
Schematic for generating brain networks from fMRI time series data (recreated from Fornito et al., 2012; Simpson et al., 2013a). Functional connectivity between brain areas is estimated based on time series pairs to produce a connection matrix. A threshold is commonly applied to the matrix to remove negative and/or “weak” connections.

Assuming fMRI connection matrices have already been obtained (see Figure 1 recreated from Fornito et al., 2012; Simpson et al., 2013a), let ***C***_***ijk***_ represent a weighted *n*_*n*_ × *n*_*n*_ connection matrix for individual *i* within-task *j* on repetition *k*, with matrix entries ranging from −1 (perfect negative correlation) to 1 (perfect positive correlation). We only considered undirected networks, so matrices were symmetric, with the *# of row* = *# of columns* = *# of nodes* (methods are adaptable if directed networks are desired).

Let ***B***_***ijk***_ represent an *n*_*n*_ × *n*_*n*_ binary graph for individual *i* within-task *j* on repetition *k*, with an entry of 1 representing a key connection and 0 a connection that is not key. Key connections are most frequently identified with thresholding, where all connections greater than a certain value get mapped to 1, while the rest get mapped to 0. Since key connections were compared across subjects, it was important to employ the same criterion in all the networks (e.g., top 10% highest connection strengths, correlation > 0.5, etc.).

It should be noted that none of the methods employed here, except for the manifold distances, are specific to differences between connection matrices. That is, these methods could also be implemented on differences between nodal degree vectors or nodal scaled inclusivity, for example.

### Step 2: Establish Similarity/Dissimilarity Between Networks

This section covers some of the metrics we used to gauge distances between individual networks given the insight they can provide into brain network organizational differences.

#### Kolmogorov–Smirnov statistic.

Degree distributions, which help quantify the topology of networks, are likely more similar within distinctive groups than they are between these groups. We again employed the log of the KS statistic to quantify this potential dissimilarity as we did for our single-task approach.

$$\text{log}\left(KS_{abc,def}\right)=\text{log}\left(sup_{x}\left|F_{abc}\left(x\right)-F_{def}\left(x\right)\right|\right)$$

*KS*_*abc*,*def*_, a scalar, is the KS statistic between connectivity matrix ***C***_***abc***_ (individual *a*, task *b*, repetition *c*) and connectivity matrix ***C***_***def***_. *F*_*abc*_(*x*) represents the empirical distribution function for observations from the off-diagonal upper (or lower) triangular portion of ***C***_***abc***_. So, *sup*_*x*_|*F*_*abc*_(*x*) − *F*_*def*_(*x*)| gives the biggest difference between the empirical edge connectivity distributions between ***C***_***abc***_ and ***C***_***def***_. Bigger values indicate more dissimilarity.

A note on the logarithmic transformation of the KS statistic: when all distances are nonnegative, it is common practice to take a log transformation. Within our simulations, KS was the only metric that saw improvements in power or type I error when taking such a transformation. For ease of interpretability, none of the other distances presented here utilized a logarithmic transformation.

#### Jaccard distance.

$${JD}_{abc,def}=\frac{M_{01}+M_{10}}{M_{11}+M_{01}+M_{10}}$$

*JD*_*abc*,*def*_, a scalar, is the Jaccard distance (JD) between binary graph ***B***_***abc***_ (individual *a*, task *b*, repetition *c*) and binary graph ***B***_***def***_. *M*_11_ is the number of off-diagonal upper (or lower) triangular connections such that ***B***_***abc***_ and ***B***_***def***_ both have a value of 1, *M*_01_ is the number of connections where ***B***_***abc***_ = 0 and ***B***_***def***_ = 1, and *M*_10_ is the number of connections where ***B***_***abc***_ = 1 and ***B***_***def***_ = 0. *JD*_*abc*,*def*_ gives the proportion of key edges (in either set) that do not share key status between ***B***_***abc***_ and ***B***_***def***_. Values of *JD*_*abc*,*def*_ range from 0 (perfect overlap) to 1 (no overlap).

#### Log-Euclidean Riemannian metric (LERM).

$${\text{LERM}}_{abc,def}={\left\|\text{log}\left(C_{abc}\right)-\text{log}\left(C_{def}\right)\right\|}_{F}$$

*LERM*_*abc*,*def*_, a scalar, is the log-Euclidean distance between connectivity matrix ***C***_***abc***_ and connectivity matrix ***C***_***def***_, where the exponential of a matrix ***A*** is defined by *e*^***A***^ ≡ $\sum_{i=0}^{\infty }$ ***A***^*n*^/*n*!. ***B*** is said to be a matrix logarithm of ***C*** if *e*^***B***^ = ***C*** (Hall, 2015); ‖·‖_*F*_, the Frobenius or Euclidean matrix norm, is defined as the square root of the sum of the absolute squares of its elements (Golub & Loan, 1996). An in-depth look at Riemannian geometry on SPD matrices and its applications for the analysis of functional connectivity can be found here (You & Park, 2021). LERM was calculated using the pdDist function in the pdSpecEst package in R (Chau, 2020).

#### Pearson correlation distance.

$${PCD}_{abc,def}=\frac{1-\text{corr}\left(C_{abc},C_{abc}\right)}{2}$$

*PCD*_*abc*,*def*_, a scalar, is the Pearson correlation distance (PCD) between connectivity matrix ***C***_***abc***_ and connectivity matrix ***C***_***def***_, where *corr*(***C***_***abc***_, ***C***_***abc***_) represents the Pearson correlation coefficient between the vectorized off-diagonal values of the two matrices. The above equation is calculated for the off-diagonal upper triangular portion of the matrices (easily adaptable to upper and lower triangular if nonsymmetric). Values of *PCD*_*abc*,*def*_ range from 0 to 1.

#### Euclidean distance (EUC).

$$E_{abc,def}=={\left(\sum_{i=1}^{n_{n}}\sum_{j=i+1}^{n_{n}\;}{\left|C_{abc}\left[i,j\right]-C_{def}\left[i,j\right]\right|}^{2}\right)}^{\frac{1}{2}}$$

*E*_*abc*,*def*_, a scalar, is the Euclidean distance between connectivity matrix ***C***_***abc***_ and connectivity matrix ***C***_***def***_, where ***C***_***abc***_[*i*, *j*] represents the connectivity matrix value (edge weight) between node *i* and node *j* for individual *a*, task *b*, and repetition *c*. The above equation is calculated for the off-diagonal upper triangular portion of the matrices (easily adaptable to upper and lower triangular if nonsymmetric). Bigger values of Euclidean distance indicate more dissimilarity.

### Step 3: Evaluating Differences Between Networks

#### Standard *F* test.

$${\text{Dist}}_{abc,dbf}=X_{abc,dbf,con}^{T}\beta_{b,con}+X_{abc,dbf,\text{coi}}^{T}\beta_{b,\text{coi}}+\varepsilon_{abc,dbf}$$

*Dist*_*abc*,*dbf*_ represents the distance between connectivity matrix ***C***_***abc***_ and connectivity matrix ***C***_***def***_ (distinct individuals *a* and *d*, same-task *b*, and all combinations of repetitions *c* and *f*). *Dist*_*abc*,*dbf*_ is a generic placeholder for any metric outlined previously in Step 2, that is, Jaccard distance (*JD*_*abc*,*dbf*_), KS statistic (*KS*_*abc*,*dbf*_), and so on.

$X_{abc,dbf,con}^{T}$, a 1 × (*p* − 1) vector, contains the intercept and differences in confounding covariates (e.g., for our data, sex, educational attainment, age, and body mass index) between distinct individuals *a* and *d*, same-task *b*, and all combinations of repetitions *c* and *f* (with corresponding unknown (*p* − 1) × 1 task *b* parameter vector ***β***_***b***,**con**_) to control for differences that may confound the relationship between the covariate of interest and the given distance.

$X_{abc,dbf,\text{coi}}^{T}$, a scalar, contains the difference in the covariate of interest (or an indicator of different group membership for group-based analyses) between distinct individuals *a* and *d*, same-task *b*, and all combinations of repetitions *c* and *f* (with corresponding unknown task *b* parameter *β*_*b*,coi_).

Splitting the design matrix ***X***_***b***_, an *n* × *p* matrix, into confounding and of interest covariates is purely a notational preference. $X_{abc,dbf,con}^{T}$ and $X_{abc,dbf,\text{coi}}^{T}$ can be combined into the 1 × *p* vector $X_{abc,dbf}^{T}$ (with corresponding unknown *p* × 1 parameter vector ***β***_***b***_).

*ε*_*abc*,*dbf*_ accounts for the random error in the distance (Jaccard, KS, etc.) value. If the random errors were independent, homoscedastic, and approximately normally distributed, the *F* test of a standard linear regression would be an appropriate test. However, here we have correlated observations, so this standard testing procedure was just included for comparison.

As an example, to test (specifically for task *b*) whether there is an association between IQ (continuous) and the spatial consistency of network edges (top 20% highest positive correlation) after controlling for age (continuous), sex (binary), and treatment (binary) status, our model would be

$${JD}_{abc,dbf}=\beta_{b,0}+\left|{Age}_{abc}-{Age}_{dbf}\right|\beta_{b,1}+𝟙\left\{{Sex}_{abc}\neq {Sex}_{dbf}\right\}\beta_{b,2}+𝟙\left\{{Trt}_{abc}\neq {Trt}_{dbf}\right\}\beta_{b,3}+\left|{IQ}_{abc}-{IQ}_{dbf}\right|\beta_{b,4}+\varepsilon_{abc,dbf}$$

with the associated null hypothesis *H*_0_ : *β*_*b*,4_ = 0.

Note that there are task-specific design matrices. The aggregated model looks as follows:

$$\text{Dist}=X^{T}\beta +\epsilon =X_{b_{1}}^{T}\beta_{b_{1}}+\ldots +X_{b_{n_{t}}}^{T}\beta_{b_{n_{t}}}+\epsilon$$

That is, we will have task-specific inference (parameter estimates, *p* values, etc.).

#### Standard *F* test with scan-level fixed effects (*F* test with SLE).

$$\text{Dist}=X^{T}\beta +{\text{SCANID}}_{1,1,1}\alpha_{1,1,1}+\ldots +{\text{SCANID}}_{np,nt,nr}\alpha_{np,nt,nr}+\epsilon$$

**Dist** is an *n* × 1 vector of known distance metrics (as outlined in Step 2). ***X***^***T***^ is the *n* × *n*_*t*_*p* design matrix (intercepts optional) of known covariates with corresponding *n*_*t*_*p* × 1 unknown parameter vector ***β***. **SCANID**_**i**,**j**,**k**_ is the *n* × 1 known indicator variable for the brain scan of individual *i*, task *j* and repetition *k*, with corresponding unknown parameter *α*_*i*,*j*,*k*_. Accounting for SLE allowed for an *F* test to appropriately evaluate the covariates of interest in our previous method (Tomlinson et al., 2022). Given that there are now repeated within-task comparisons between distinct individuals, we do not expect this approach to still render the *F* test appropriate. This testing procedure was included here mainly for comparison and to highlight that multiple brain scans per individual will require additional considerations.

#### Mixed model for multitask (and multisession) BrAin NeTwOrk regression (3M_BANTOR).

$$\text{Dist}=X^{T}\beta +{\text{SCANID}}^{T}\alpha +\epsilon +{ID}_{1,1}\_{ID}_{2,1}b_{1,1;2,1}+{ID}_{1,1}\_{ID}_{3,1}b_{1,1;3,1}+\ldots +{ID}_{1,1}\_{ID}_{n_{p},1}b_{1,1;n_{p},1}+{ID}_{2,1}\_{ID}_{3,1}b_{2,1;3,1}+{ID}_{2,1}\_{ID}_{4,1}b_{2,1;4,1}+\ldots +{ID}_{2,1}\_{ID}_{n_{p},1}b_{2,1;n_{p},1}+\ldots +{ID}_{n_{p}-1,n_{t}}\_{ID}_{n_{p},n_{t}}b_{n_{p}-1,n_{t};n_{p},n_{t}}$$

**Dist** is an *n* × 1 vector of known distance metrics (as outlined in Step 2). ***X***^***T***^***β*** and **SCANID**^***T***^***α*** are combined versions of what was outlined in the previous section. ***ID***_***a*,*b***__***ID***_***d*,*b***_ is the *n* × 1 known indicator variable for comparisons between distinct individuals *a* and *d* on same-task *b*, with *b*_*a*,*b*;*d*,*b*_ ∼ *N*(0, *g*_*b*_) being the corresponding random effect. Linear mixed effects modeling was done with REML using the lmer function of the R package lme4 (Bates et al., 2015); *p* values were calculated using Satterthwaite’s method (Fai & Cornelius, 2007) using the R package lmerTest (Kuznetsova et al., 2020).

Please note, if one attempted to use 3M_BANTOR with single-task cross-sectional data (i.e., just one task and no repeated scans per subject), there would be one random effect for each observation (and your statistical software of choice would likely report an error or warning telling you something along the lines of “the number of levels of a grouping factor for the random effects must be less than the number of observations”). In this case, all random effects should be dropped, and 3M_BANTOR effectively turns into the model shown in the previous section, a standard *F* test with scan-level fixed effects. This is equivalent to the recommended testing method from our previous method (Tomlinson et al., 2022).

#### MDMR permutation and mixed-MDMR.

Multivariate distance matrix regression (MDMR) is an existing method that has been included here for comparison. It tests the significance of associations of response profile (dis)similarities and a set of predictors. Originally this was done using only permutation tests (Anderson, 2001), but has been extended to analytic *p* values and nonindependent observations (McArtor, 2017). We ran both MDMR permutation and mixed-MDMR (for nonindependent observations) methods.

For our previously mentioned methods, distances were limited to within-task comparisons. Since MDMR methods require a complete distance matrix, we instead ran MDMR separately for each task. Additionally, in the above methods, observations were limited to distinct individuals. For similar reasoning (methods require a complete distance matrix), this is not possible in the MDMR framework and within-individual distances were included. Inputs into each (individual-task) model were with the *n*_*p*_*n*_*r*_ × *n*_*p*_*n*_*r*_ distance matrix ***D*** (the distance matrix analog of **Dist**) and the *n*_*p*_*n*_*r*_ × *p* design matrix ***X***_***p***_ (covariates of interest for each participant).

MDMR permutation was run using the mdmr function in the MDMR package in R (McArtor, 2018) using the permutation method with 5,000 permutations. This method does not account for correlation among individuals and was included mainly for comparison. Mixed-MDMR accounts for nonindependent observations, and was run with individual-level random intercepts (analogous to section Mixed model for multitask (and multisession) BrAin NeTwOrk regression (3M_BANTOR)) using the mixed.mdmr function in the MDMR package in R (McArtor, 2018). In its current form, mixed-MDMR does not allow for mixed level models. That is, random intercepts can be included at the individual level or at the group level, but not both.

For a summarization of why previous methods are not suitable methods for relating covariates to distances in the multitask and multisession context, please see Table 1.

**Table 1.** A summarization of why previous methods are not suitable methods for relating covariates to distances in the multitask and multisession context

**Method**	**Limitation in the multitask and multisession context**
***F* test**	Correlated observations
***F* test with SLE**	Repeated within-task comparisons between distinct individuals
**MDMR**	Requires complete distance matrix, which means assessing the distance across task/rest connectomes

## SIMULATION STUDIES

The following simulation study is done using a factorial approach. There are three different task states (named Tasks 1–3). For each simulation setting, we explore four different metrics (KS, JD, EUC, and LERM). For each task and metric combination, five different methods are considered (*F* test, *F* test SLE, 3M_BANTOR, MDMR permutation, and mixed-MDMR). Subsequent sections will present details and results for each of these “factors.” (Note: *F* test, *F* test SLE, and 3M_BANTOR are run with all three tasks included in each method. Both MDMR methods are run separately for each Task. See Methods section for more information.)

### Data

We varied simulation settings to assess how well our proposed approaches could detect relationships between brain network properties and covariates of interest. Each simulation contained 100 subjects, with four covariates of interest. A fair coin was flipped for each subject to determine their sex (*SEX* = male or female) and treatment status (TRT = treatment or placebo). IQ and Age were both simulated from a normal distribution with mean of 100 and a standard deviation of 15 (rounded to the nearest integer). This resulted in two binary (*SEX*, *TRT*) and two continuous (*AGE*, *IQ*) covariates—variables were given names purely for purposes of explication.

We simulated fMRI connectivity matrices with 268 nodes to mimic the experimental data detailed in the next section. In each simulation, 12 (4 repetitions for each of the 3 tasks) 268 × 268 symmetric matrices (with entries ranging from −1 to 1) were generated for each subject.

Time series of 2,500 points (to mimic fMRI BOLD signal) were simulated for each node and were drawn from three types of distributions:

1. A low-connectivity noise distribution: low-connectivity noise nodes were drawn from a *Normal*(**0**, **Σ**_**1**_) where **0** is the 268 × 1 0-vector, and **Σ**_**1**_ is the 268 × 268 random correlation matrix detailed below.
  Defining **Σ**_**1**_: Let ***Q*** be the 268 × 268 random orthonormal matrix generated using methods based on a QR decomposition (Mezzadri, 2007). This was done using the randortho function in the pracma package in R (Borchers, 2021). Let ***D*** be the 268 × 268 diagonal matrix with diagonal entries simulated from a *Beta*($\frac{3}{4}$, 2) multiplied times 50. Let **Σ**_**1**_ = ***B***^−**1**/**2**^***AB***^−**1**/**2**^ where ***A*** = ***Q***^***T***^***DQ*** and ***B*** is the 268 × 268 diagonal matrix with diagonal entries matching the diagonal of ***A***. Finally, correlation smoothing (Bock et al., 1988; Wothke, 1993) was done on **Σ**_**1**_ using the cor_smooth function in the correlation package in R (Makowski et al., 2022) which utilizes the cor.smooth function in the psych package in R (Revelle, 2021). Tolerance for correlation smoothing was chosen to be 10^−6^.
2. A high-connectivity noise distribution: high-connectivity noise nodes were drawn from a *Normal*(**0**, **Σ**_**2**_) where **0** is the 15 × 1 0-vector and **Σ**_**2**_ is the 15 × 15 correlation matrix with 1’s down the diagonal and all off-diagonal entries equal a single draw from a *Beta*(5, 5) distribution.
3. A signal distribution dependent on covariates and signal percentage: signal and covariate-dependent nodes were drawn from a *Normal*(**0**, **Σ**_**3**_) where **0** is the 15 × 1 0-vector and **Σ**_**3**_ is the 15 × 15 correlation matrix with 1’s down the diagonal. All off diagonals equal a single draw from a (1 − *s*_*p*_) · *Sample*(**Σ**_**1**_) + *s*_*p*_ · *Beta*(*a*_*i*_, 15) distribution where *a*_*i*_ = *min* (5.95, *max* (−5.95, (*IQ*_*i*_ − 100) * .15 + (*Trt*_*i*_ == “*Treatment*”) * 2 − (*Trt*_*i*_ == “*Placebo*”) * 2)) represented the covariate-dependent parameter, *s*_*p*_ represented the signal percentage (from 0 to 100%), and *Sample*(**Σ**_**1**_) represented a random draw from the off-diagonal of **Σ**_**1**_. When the signal percent (*s*_*p*_) was 100%, (1 − *s*_*p*_) · *Sample*(**Σ**_**1**_) + *s*_*p*_ · *Beta*(7 + *a*_*i*_, 7 − *a*_*i*_) = *Beta*(7 + *a*_*i*_, 7 − *a*_*i*_). Similarly, when signal percent was 0%, (1 − *s*_*p*_) · *Sample*(**Σ**_**1**_) + *s*_*p*_ · *Beta*(*a*_*i*_, 15) = *Sample*(**Σ**_**1**_), and was therefore identical to the low-connectivity noise distribution and no longer dependent on covariates.

Each Task had three 15-node regions from either the high-connectivity noise distribution or a covariate-dependent signal distribution. Task 1 had two 15-node regions (node regions x and y) where all individuals had the same high-connectivity noise distribution, one 15-node region (node region z) where the signal distribution was dependent on covariates. Task 2 had one 15-node region (x) where all individuals had the same high-connectivity noise distribution and two 15-node regions (y and z) where the signal distribution was dependent on covariates. Task 3 had three 15-node regions (x, y, and z) where the signal distribution was dependent on covariates. Each node region was correlated in the following way: 12 quantiles (four repetitions for each of the three tasks) were drawn from a standard multivariate-normal distribution with a 12 × 12 covariance matrix: 1’s down the diagonal, 0.7 for within-task, 0.3 for within-repetition, and 0 otherwise (see Table 2). These quantiles were then used to draw from either the high-connectivity noise distribution or the signal distribution dependent on covariates and signal percentage detailed in the preceding paragraphs.

**Table 2.** Within- and between-task correlation table used for simulations

		Task 1				Task 2				Task 3			
		Rep. 1	Rep. 2	Rep. 3	Rep. 4	Rep. 1	Rep. 2	Rep. 3	Rep. 4	Rep. 1	Rep. 2	Rep. 3	Rep. 4
Task 1	Rep. 1	1	0.7	0.7	0.7	0.3	0	0	0	0.3	0	0	0
	Rep. 2	0.7	1	0.7	0.7	0	0.3	0	0	0	0.3	0	0
	Rep. 3	0.7	0.7	1	0.7	0	0	0.3	0	0	0	0.3	0
	Rep. 4	0.7	0.7	0.7	1	0	0	0	0.3	0	0	0	0.3
Task 2	Rep. 1	0.3	0	0	0	1	0.7	0.7	0.7	0.3	0	0	0
	Rep. 2	0	0.3	0	0	0.7	1	0.7	0.7	0	0.3	0	0
	Rep. 3	0	0	0.3	0	0.7	0.7	1	0.7	0	0	0.3	0
	Rep. 4	0	0	0	0.3	0.7	0.7	0.7	1	0	0	0	0.3
Task 3	Rep. 1	0.3	0	0	0	0.3	0	0	0	1	0.7	0.7	0.7
	Rep. 2	0	0.3	0	0	0	0.3	0	0	0.7	1	0.7	0.7
	Rep. 3	0	0	0.3	0	0	0	0.3	0	0.7	0.7	1	0.7
	Rep. 4	0	0	0	0.3	0	0	0	0.3	0.7	0.7	0.7	1

*Note*. Each node region (x, y, and z) was correlated in the following way: 12 quantiles (four repetitions for each of the three tasks) were drawn from a standard multivariate-normal distribution with a 12 × 12 covariance matrix: 1’s down the diagonal, 0.7 for within-task, 0.3 for within-repetition, and 0 otherwise. These quantiles were then used to draw from either the high-connectivity noise distribution or the signal distribution dependent on covariates and signal percentage detailed in the proceeding paragraphs.

The remaining time series for nodes from all tasks were directly drawn from the low-connectivity noise distribution. Pearson correlation matrices were then calculated from the simulated time series and smoothed using the cor_smooth function in the correlation package in R (Makowski et al., 2022) which utilizes the cor.smooth function in the psych package in R (Revelle, 2021). Tolerance for correlation smoothing was chosen to be 10^−6^.

**Figure 2.**
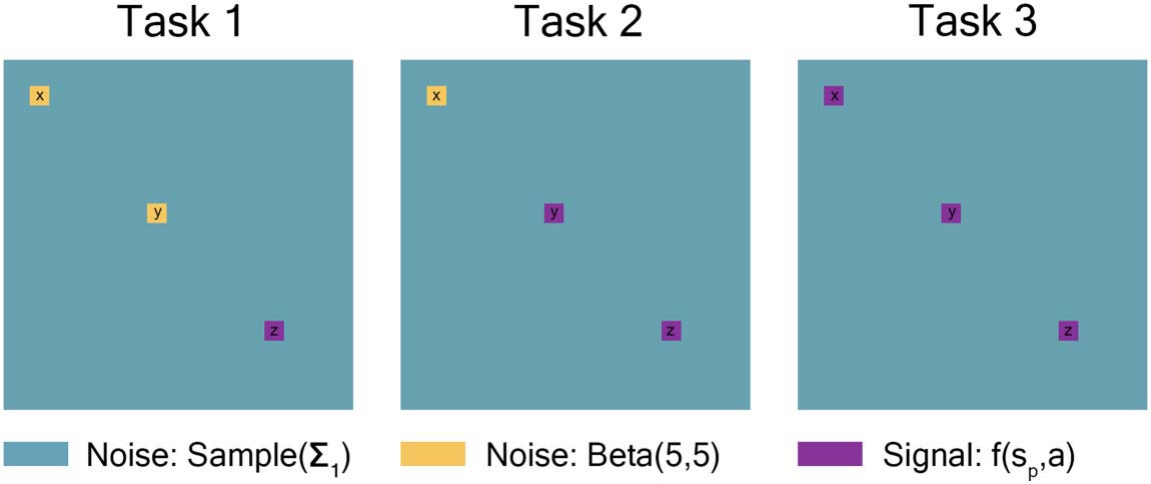
Task 1 had two 15-node regions where all individuals had the same high-connectivity noise distribution, and one 15-node region where the signal distribution was dependent on covariates. Task 2 had one 15-node region where all individuals had the same high-connectivity noise distribution and two 15-node regions where the signal distribution was dependent on covariates. Task 3 had three 15-node regions where the signal distribution was dependent on covariates. The remaining nodes from all tasks were drawn from the low-connectivity noise distribution. It should be noted that connectivity matrices are symmetric, with entries of 1 along the diagonal. Further, low-connectivity noise (teal) entries along the rows and columns of high-connectivity noise (yellow) and signal-dependent (purple) regions will be affected by those “yellow” and “purple” entries. This figure is drawn to scale.

For a drawn to scale representation of these simulations, see Figure 2. It should be noted that connectivity matrices are symmetric, with entries of 1 along the diagonal. Further, low-connectivity noise (teal) entries along the rows and columns of high-connectivity noise (yellow) and signal-dependent (purple) regions will be affected by those “yellow” and “purple” entries.

**Figure 3.**
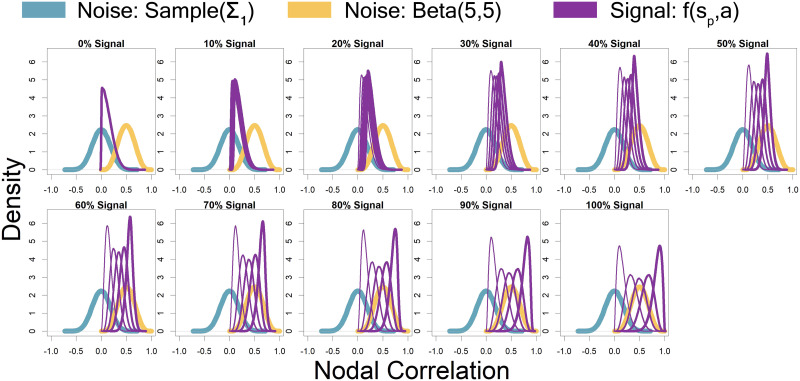
Represented by the same colors from Figure 2 simulated connectivity matrices, Figure 3 displays the distributions used for those matrices as signal percentage increased. The low-connectivity noise distribution was distributed *Sample*(**Σ**_**1**_), which represents a random draw from the off-diagonal of a random correlation matrix **Σ**_**1**_, and was shown in teal (not affected by signal percentage). The high-connectivity noise distribution was distributed Beta(5,5) and is in yellow (not affected by signal percentage). The “covariate-dependent” signal region can be seen in purple and was distributed (1 − *s*_*p*_) · *Sample*(**Σ**_**1**_) + *s*_*p*_ · *Beta*(*a*, 15). The *a* parameter had some distribution based on the underlying covariate distribution and signal percentage. There are five purple distributions in each plot representing the 0.05, 0.25, 0.5, 0.75, and 0.95 quantiles (shown in increasing thickness) from the *a* distribution (for example, the 0.25 quantile distribution is represented by an individual with an IQ of 70 and a “Treatment” status or an individual with an IQ of 100 and a “Placebo” status; the 0.75 quantile distribution is represented by an individual with an IQ of 100 and a “Treatment” status or an individual with an IQ of 130 and a “Placebo” status). Further, the “covariate-dependent” (purple) signal region’s distribution goes from being the same as the absolute value of the low-connectivity noise region’s distribution (at 0% signal) to more and more different than the noise region’s distribution as signal percentage increases.

Represented by the same colors from Figure 2 simulated connectivity matrices, Figure 3 displays the distributions used for those matrices as signal percentage increased. The low-connectivity noise distribution was distributed *Sample*(**Σ**_**1**_), which represents a random draw from the off-diagonal of a random correlation matrix **Σ**_**1**_, and was shown in teal (not affected by signal percentage). The high-connectivity noise distribution was distributed Beta(5, 5) and is in yellow (not affected by signal percentage). The “covariate-dependent” signal region can be seen in purple and was distributed (1 − *s*_*p*_) · *Sample*(**Σ**_**1**_) + *s*_*p*_ · *Beta*(7 + *a*, 7 − *a*). The *a* parameter had some distribution based on the underlying covariate distribution and signal percentage. There are five purple distributions in each plot representing the 0.05, 0.25, 0.5, 0.75, and 0.95 quantiles (shown in increasing thickness) from the *a* distribution (for example, the 0.25 quantile distribution is represented by an individual with an IQ of 70 and a “Treatment” status or an individual with an IQ of 100 and a “Placebo” status; the 0.75 quantile distribution is represented by an individual with an IQ of 100 and a “Treatment” status or an individual with an IQ of 130 and a “Placebo” status). Further, the “covariate-dependent” (purple) signal region’s distribution goes from being the same as the absolute value of the low-connectivity noise region’s distribution (at 0% signal) to more and more different than the noise region’s distribution as signal percentage increases.

### Results

We assessed methods with 2,500 simulations as detailed in the previous section. Key connections of interest (binary graphs used for the Jaccard distance) based on edge correlation were identified, selecting the top 20% and top 0.05% highest (positive) correlations and mapping those to 1 while mapping all remaining edges to 0. The KS statistic, LERM, PCD, and Euclidean distance were calculated for each pair of same-task connectivity matrices. The Jaccard distance was calculated for each pair of same-task binary graphs. MDMR methods requires inclusion of same-individual comparisons, while all other methods throw these comparisons out.

**Figure 4.**
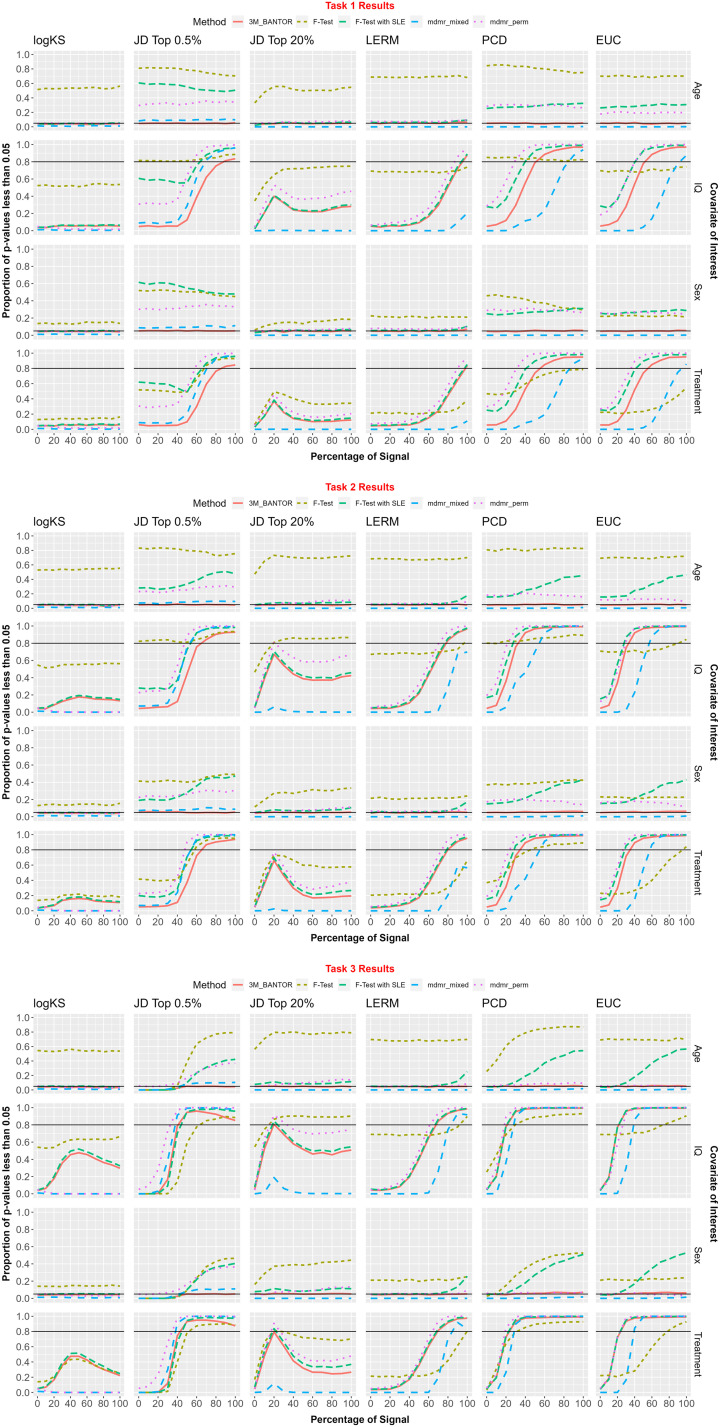
We assessed methods with 2,500 simulations, as detailed in the previous section. The percentages of *p* values less than *α* = 0.05 for the covariates of interest were recorded for each combination of signal percent (0%, 10%, …, 100%), distance metric (KS, Jaccard distance Top 0.5%, Jaccard distance Top 20%, log-Euclidean Riemannian metric, Pearson correlation distance, Euclidean), and testing framework (*F* test, *F* test with scan-level effects, 3M_BANTOR, MDMR permutation, and MDMR-mixed). It should be noted here that age and sex are “null” covariates (that have no bearing on the data generating process) and are included to assess type I error control of the methods on both continuous and categorical variables. Black horizontal lines are shown at 5% and 80% for aid in referencing type I error and power, respectively.

The percentages of *p* values less than *α* = 0.05 for the covariates of interest were recorded for each combination of signal percent (0%, 10%, …, 100%), distance metric (KS, JD, EUC, and LERM), and testing framework (*F* test, *F* test with SLE, 3M_BANTOR, MDMR Permutation, and MDMR-mixed). In this section, we discuss whether type I error rate was controlled and at what signal percent the 80% power threshold was reached. For a visual display of the results, see Figure 4.

The standard *F* test, *F* test with SLE, and MDMR permutation did not control type I error when testing age and sex. They were included in the figure for reference, but not mentioned any further in this section. Thus, the following is a comparison among the methods 3M_BANTOR and MDMR-mixed.

#### Kolmogorov–Smirnov.

For the KS metric, 3M_BANTOR adequately controlled type I error while MDMR-mixed had type I error close to 0. Task 1–3: neither method reached 80% power on continuous or binary covariates.

#### Jaccard top 0.5%.

For the Jaccard top 0.5% metric, 3M_BANTOR adequately controlled type I error while MDMR-mixed did not control type I error for either Age or Sex. Therefore, we only discuss power for 3M_BANTOR here. Task 1: 3M_BANTOR reached the power threshold at 90% of signal for continuous and binary covariates. Task 2: 3M_BANTOR reached the power threshold at 70% of signal for continuous and binary covariates. Task 3: 3M_BANTOR reached the power threshold at 50% of signal for continuous and binary covariates.

#### Jaccard top 20%.

For the Jaccard top 20% metric, 3M_BANTOR adequately controlled type I error while MDMR-mixed had type I error close to 0. Task 1 and 2: neither method reached the power threshold for continuous nor binary covariates. Task 3: 3M_BANTOR reached the power threshold at 20% of signal for continuous and binary covariates but fell back below the threshold as signal increased. MDMR-mixed never reached the power threshold.

#### LERM.

For the LERM metric, 3M_BANTOR adequately controlled type I error while MDMR-mixed had type I error close to 0. Task 1: 3M_BANTOR reached the power threshold at 100% of signal for continuous and binary covariates. MDMR-mixed did not reach the power threshold. Task 2: 3M_BANTOR reached the power threshold at 80% of signal for continuous and binary covariates. MDMR-mixed did not reach the power threshold. Task 3: 3M_BANTOR reached the power threshold at 70% of signal for continuous and binary covariates. MDMR-mixed reached the power threshold at 90% of signal for continuous and binary covariates.

#### Pearson correlation distance.

For the PCD, 3M_BANTOR adequately controlled type I error while MDMR-mixed had type I error close to 0. Task 1: 3M_BANTOR reached the power threshold at 60% of signal for continuous and binary covariates. MDMR-mixed reached the power threshold at 100% of signal for continuous and binary covariates. Task 2: 3M_BANTOR reached the power threshold at 40% of signal for continuous and binary covariates. MDMR-mixed reached the power threshold at 60% of signal for continuous and binary covariates. Task 3: 3M_BANTOR and MDMR-mixed reached the power threshold at 30% of signal for continuous and binary covariates.

#### Euclidean.

For the Euclidean metric, 3M_BANTOR adequately controlled type I error while MDMR-mixed had type I error close to 0. Task 1: 3M_BANTOR reached the power threshold at 60% of signal for continuous and binary covariates. MDMR-mixed reached the power threshold at 90% of signal for continuous and binary covariates. Task 2: 3M_BANTOR reached the power threshold at 40% of signal for continuous and binary covariates. MDMR-mixed reached the power threshold at 60% of signal for continuous and binary covariates. Task 3: 3M_BANTOR reached the power threshold at 30% of signal for continuous and binary covariates. MDMR-mixed reached the power threshold at 40% of signal for continuous and binary covariates.

## EXPERIMENTAL STUDIES

### Data

The fMRI data used for this project come from the HCP Young Adult 1,200 subjects Minimally Processed Data Release (Van Essen et al., 2013). Subjects were selected from the Retest subset to include a second set of fMRI scan data. Each subject completed two scan sessions. At each session, resting-state and working memory fMRI data were collected, in addition to other HCP tasks that are not used here. Two scans were collected sequentially for each paradigm with different phase encoding (right to left and left to right). After quality checks of head motion and the minimal processing, we had 45 subjects with these scans available. The HCP dataset contains subjects belonging to the same family group. To ensure between-subject independence, we performed a random selection of one subject in each family. This left us with 26 subjects used for our analyses.

The resting-state paradigm had participants quietly view a fixation cross. The working memory paradigm had participants complete randomized 0-back and 2-back tasks in a paired block design interleaved with a rest block. The working memory blocks were also randomized with photos from one of four types (faces, body parts, houses, and tools). Prior to each block, participants were alerted to the format of the block. Our analyses only used the 2-back blocks from this paradigm as described below. During the 2-back, participants were instructed to respond if the current stimulus matched the stimulus two trials back. Both the fMRI paradigms were collected as blood oxygenation level–dependent (BOLD)-weighted images with TR = 720 ms, TE = 33.1 ms, voxel size 2 mm^3^, 72 slices. The resting state collected 1,200 volumes and the working memory collected 405 volumes.

The main covariate of interest for this analysis was fluid intelligence. Other covariates included in the model formulation were age, BMI, education, handedness, income, race, sex, and smoking status (alcohol abuse, alcohol dependence, and ethnicity were left out due to homogeneity). For a summarization and explanation of the variables, see Table 3 and Table 4.

**Table 3.** Summarization and explanation of HCP covariates treated as continuous (within the regression framework)

	Mean (*SD*)	Notes
Age	30.2 (3.3)	In Years
BMI	26.7 (6.1)	Body Mass Index
Education	15.2 (1.8)	Integer Values 11 to 17 (years pf education completed)
Fluid intelligence	15.1 (5.4)	Integer valued from 4 to 24
Handedness	58.1 (57.2)	Values range from −100 to 100 by 5 (−100, −95, …, 95, 100)
Income	4.8 (2.3)	SSAGA income score - Total household income: <$10,000 = 1, 10K–19,999 = 2, 20K–29,999 = 3, 30K–39,999 = 4, 40K–49,999 = 5, 50K–74,999 = 6, 75K–99,999 = 7, > = 100,000 = 8

**Table 4.** Summarization and explanation of HCP covariates treated as categorical (within the regression framework)

Alcohol abuse	**1** met the DSM4 criteria for alcohol abuse, **25** did not
Alcohol dep.	**0** met the DSM4 criteria for alcohol dependence, **26** did not
Ethnicity	**26** Not Hispanic/Latino
Race	**1** Asian/Nat. Hawaiian/Other Pacific Is., **4** Black or African Am., **21** White
Sex	**17** Female, **9** Male
Smoking status	**3** reported as still smoking, **25** did not

### Data Processing and Network Generation

The Minimally Processed Data Release (Van Essen et al., 2013) was used here. Additional processing included the removal of the first 14 volumes from each scan, ICA-Aroma (Pruim et al., 2015) for motion correction, and band-pass filtering (0.009–0.08 Hz). The two scans collected at different phase encodings were then concatenated, and a regression was preformed to account for the effects of the two concatenated scans, whole-brain average signals by tissue (gray matter, white matter, and cerebral spinal fluid), as well the realignment parameters and their derivatives. Additional work was needed for the working memory scans. It was necessary to account for the block design of the task, so we modeled the design in SM12 resulting in regressors for the 0-back and rest blocks and the cues before every block. These regressors were added to the regression analysis previously mentioned. After regression, the residual signal was only kept for volumes that aligned with the 2-back block design. The blocks were then concatenated, resulting in a time series of 274 volumes. The resulting resting-state time series contained 2,372 volumes. We averaged the signal from all voxels within each region from the Shen Atlas (Shen et al., 2013) to create a 268-node time series for each scan. Functional networks were constructed for each participant by computing the Pearson (full) correlation between the resultant time series for each region pair.

### Results

Key edges of interest (binary connection matrices used for the Jaccard distance) based on correlation were identified, selecting the top 0.05% highest and mapping those to 1 while mapping all remaining edges to 0. The KS statistic, LERM, and Euclidean distance were calculated for each pair of scans by using their connection matrices. The Jaccard distance was calculated for each pair of individuals by using their binary graphs.

Distance covariates for each pair of individuals were calculated. A continuous variable’s distance (age, for instance) was calculated as |*Age*_*i*_ − *Age*_*j*_| for the pair of individuals *i* and *j*. A binary or categorical variable’s distance (Education, for instance) was calculated as 𝟙{*Edu*_*i*_ ≠ *Edu*_*j*_} for the pair of individuals *i* and *j*.

We evaluated differences between networks with our proposed 3M_BANTOR approach. Resting-state fMRI were compared between all individuals for both sessions (1 and 2) and phases (LR and RL). Working memory block design was different between the RL and LR phases, so we did not compare working memory connection matrices between phases. Thus, covariates were estimated for resting state (combining both phases), working memory (phase LR), and working memory (phase RL). Parameter and standard error estimates can be found in Supporting Information Tables S1, S2, and S3. Each parameter estimate represented the average amount the given brain distance metric (KS, Jaccard, etc.) changed based on a one-unit difference in the respective covariate, after controlling for other covariates. A complete list of *p* values for both resting state and working memory can be seen in Table 5. Given the high degree of dependence between these results, and the illustrative and exploratory nature of our analysis, there have been no adjustments for multiple comparisons.

**Table 5.** *P* values for HCP resting-state and working memory brain scans when modeled with our given regression framework and tested using the standard *F* test with fixed individual-level effects

	Resting State							
	KS		JAC		EUC		LERM	
FluidIntl	4.87E−01		6.80E−01		4.08E−01		6.98E−01	
Age	4.31E−01		6.35E−01		9.35E−01		4.57E−01	
BMI	9.90E−01		8.43E−01		4.14E−01		4.34E−01	
Education	4.80E−01		4.88E−01		4.27E−01		2.51E−01	
Gender	7.73E−01		1.92E−01		2.53E−01		1.40E−01	
Handedness	2.39E−01		6.93E−02		7.35E−01		2.92E−01	
Income	5.06E−01		7.08E−01		7.51E−01		4.71E−01	
Race	8.68E−01		7.85E−02		3.10E−03		1.08E−02	
SmokeStatus	9.11E−01		4.25E−01		6.40E−01		2.88E−01	

	Working Memory (Phase LR)				Working Memory (Phase RL)			
	KS	JAC	EUC	LERM	KS	JAC	EUC	LERM
FluidIntl	8.61E−01	3.47E−01	1.93E−01	1.32E−02	7.45E−01	8.44E−01	2.26E−01	3.29E−01
Age	1.74E−01	1.02E−01	1.19E−01	7.29E−01	7.50E−01	3.41E−01	9.84E−01	1.85E−01
BMI	4.82E−01	1.32E−01	1.82E−01	3.27E−01	5.50E−01	2.72E−02	7.23E−02	2.20E−02
Education	3.34E−01	8.47E−01	7.72E−01	7.04E−01	6.57E−01	5.08E−01	2.86E−01	8.88E−01
Gender	8.45E−02	4.94E−01	3.50E−01	4.39E−01	8.67E−01	9.38E−01	3.70E−01	4.26E−01
Handedness	6.62E−02	8.14E−01	5.04E−01	9.61E−01	9.84E−01	5.72E−01	4.02E−02	8.26E−01
Income	8.89E−01	7.67E−01	6.05E−01	4.20E−01	4.67E−01	7.19E−01	7.11E−01	8.81E−01
Race	7.93E−01	2.12E−01	3.10E−01	6.36E−01	9.18E−01	9.04E−01	1.10E−02	1.51E−02
SmokeStatus	9.97E−01	7.05E−01	4.77E−01	6.42E−01	5.30E−01	2.60E−01	8.36E−01	7.84E−02

Legend:	**0**		**0.05**				**1**	

*Note*. Parameter estimates and standard errors can be found in the Supporting Information.

After adjusting for the other confounding variables, the covariate of interest, fluid intelligence, had a statistically significant relationship for LERM during working memory (Phase LR), but did not have a statistically significant relationship with any other distance metric (KS, JAC, LERM, EUC) for resting-state or working memory fMRI.

In the Supporting Information, we show the 3M_BANTOR approach using nodal degree vectors rather than connectivity matrices. Fluid intelligence had a statistically significant relationship for Jaccard distance (top 20%) during working memory (Phase RL), but did not have a statistically significant relationship with any other distance metric (KS, Jaccard distance (top 5%), Euclidean) for resting-state or working memory fMRI when distances were calculated using nodal degree vectors (see Supporting Information).

## DISCUSSION

Our previous work developed a novel analytic framework to assess the relationship between brain network architecture and phenotypic differences while controlling for confounding variables (Tomlinson et al., 2022). More specifically, this innovative regression framework related distances (or similarities) between brain network features from a single task to functions of absolute differences in continuous covariates and indicators of difference for categorical variables. Here we extended that work to the multitask and multisession context to allow for multiple brain networks per individual, and explored several similarity metrics for comparing distances between connection matrices. While our previous work summed over the rows of connection matrices to create and show the utility of comparing nodal degree vectors, this work focused solely on the utility of distance metrics using entire connection matrices. This changed the interpretation of what a difference meant, that is, switching the individual comparisons from nodal degrees to edge weights. However, all metrics discussed here (except for LERM as it requires SPD matrices), are able to handle nodal degree vectors as well. Additionally, examining the entire connection matrix allows assessing how more global/systemic properties of networks are related to covariates, as distinct from node- or edge-based analyses (Simpson & Laurienti, 2016). The KS statistic measures how different distributions of topological properties vary between two individuals. Key-node metrics (like the Jaccard distance) quantify how much the spatial location of key brain network edges differ between two networks. The PCD and Euclidean norm measure whether the spatial location of degree-weighted brain network edges differ. The log-Euclidean Riemannian metric (LERM) is used as a computationally friendly approximation of the affine-invariant Riemannian metric (AIRM). Riemannian metrics are used to measure representational connectivity and “captures representational relationships more accurately when there are relatively small number of response channels (e.g., voxels)” (Shahbazi et al., 2021). Many other distances or similarity metrics could be used. Future work might include testing other metrics and taking a deeper dive into understanding when and how to choose a distance metric.

Several standard methods for estimation and inference were adapted to fit into our regression framework: standard *F* test, *F* test with scan-level effects (ILE), MDMR permutation, mixed-MDMR, and our proposed 3M_BANTOR approach. All combinations of these approaches and the distance metrics were assessed via three simulation scenarios. The KS statistic was found to have low power (relative to the other distance metrics) in all our simulations as we tested location-specific differences only for connection matrices. Our previous work has shown if one is interested in comparing nodal degree distributions, the KS statistic is preferred. The Jaccard top 20% distance did not have consistent or predictable power. This was due to the percentage of signal-dependent edges being considerably less than 20% (covariate-dependent signal was present in approximately 0.3% of edges in Task 1, 0.6% in Task 2, and 0.9% in Task 3), and, as signal percentage increased, most signal-dependent edges ended up in the top 20% (which were mapped to 1). If low signal edges have greater values than the noise, and the top percentage is high enough to contain all signal-dependent edges, then the Jaccard distance cannot differentiate between high and low signal connections. To account for the percentage of signal-dependent edges being considerably less than 20%, the Jaccard top 0.5% distance was explored and had consistent and predictable power as there was a good spread of signal-dependent edges both in and out of the key-node set (covariate-dependent signal was present in approximately 0.3% of edges in Task 1, 0.6% in Task 2, and 0.9% in Task 3). Two very different choices of thresholding for the Jaccard metric were chosen here to highlight that the threshold does matter and that thought should be put into what an appropriate threshold should be for the given context. Further, we should note that a top percent is only one type of thresholding; several other approaches have been used in the literature, but there is no consensus on the best approach (Simpson et al., 2013a). In our simulations (testing location-based differences), the Pearson correlation and Euclidean distances had the best combination of type I error control and power (unsurprising considering our previous work and the nature of our simulation method). Riemannian metrics look to capture several types of differences within functional connectivity, and the LERM metric showed in our simulations that it does capture location-specific distances well. As evidenced by the variety of results here, future work should include a further investigation into how and when to choose specific distance metrics. The “best” metric to choose in this framework will largely depend on the types of differences one is looking to detect.

Regarding the comparison of estimation and testing methods (standard *F* test, *F* test with SLE, etc.), our proposed 3M_BANTOR approach was the only method to control type I error across all metrics. The standard *F* test, as in our previous work, was not able to control type I error in a distance regression framework. *F* test with SLE and MDMR permutation, methods that worked well in our previous work, were not able to control type I error well when multiple scans were present for each individual. MDMR-mixed did not control type I error when testing the Jaccard top 20%. MDMR-Mixed with other metrics had the strange property we noticed with MDMR in our previous work; it had a type I error rate near 0. As for power, our proposed 3M_BANTOR beat MDMR-mixed across all metrics in our simulations.

An analysis of the HCP data was completed using 3M_BANTOR and several distance metrics. After adjusting for the other confounding variables, the covariate of interest, fluid intelligence, had a statistically significant relationship for LERM during working memory (Phase LR), but did not have a statistically significant relationship with any other distance metric (KS, Jaccard distance, Euclidean, LERM) for resting-state or working memory fMRI when distances were calculated using connectivity matrices. Fluid intelligence, had a statistically significant relationship for Jaccard distance (top 20%) during working memory (Phase LR), but did not have a statistically significant relationship with any other distance metric (KS, Jaccard distance (top 5%), Euclidean) for resting-state or working memory fMRI when distances were calculated using nodal degree vectors (see Supporting Information). This is somewhat counter to our previous work, and could be due to power issues from a low sample size or the different (possibly noisier) set of processed data. Extending our approach to account for familial correlation would allow increasing our sample size with the HCP data and is planned for future work. Moreover, it is important to note that this modeling approach provides unique and complementary insight to others, and thus the properties it examines may in fact not be related to covariates that other network properties may be related to.

Our methodology has applicability to a wide set of clinical populations. For example, connectome-wide association studies have become very popular, where regions are identified by the statistical association of their whole-brain patterns with given phenotypic traits (Shehzad et al., 2014). This has been implemented mainly using MDMR and successfully applied to different pathological populations (Alzheimer’s disease, schizophrenia, etc.) and using different brain network measures (e.g., structural connectivity). A few recent examples include common and dissociable mechanisms of executive system dysfunction across psychiatric disorders in youth (Shanmugan et al., 2016), cerebellar-prefrontal network connectivity and negative symptoms in schizophrenia (Brady et al., 2019), group-level progressive alterations in brain connectivity patterns revealed by diffusion-tensor brain networks across severity stages in Alzheimer’s disease (Rasero et al., 2017a), connectome-wide investigation of altered resting-state functional connectivity in war veterans with and without posttraumatic stress disorder (Misaki et al., 2018), multivariate regression analysis of structural MRI connectivity matrices in Alzheimer’s disease (Rasero et al., 2017b), and aberrant temporal connectivity in persons at clinical high risk for psychosis (Colibazzi et al., 2017). Our regression framework could also be used for connectome-wide association studies in a manner similar to those studies described above, with the advantage of being in principle more suitable than MDMR, as this article shows. It is also important to note that our regression framework can accommodate any type of connectome data—for example, structural or functional connectomes from other modalities like EEG and MEG—and data from different atlases, providing additional flexibility. One could also average the distance for the rest and task conditions to take advantage of shared features (Elliott et al., 2019; Gao et al., 2019), but it is also possible that network features unique to one condition may be specifically associated with the study variables of interest while the other may not. Thus, more work needs to be done in this area, and it is certainly possible that the validity of averaging over conditions is a study-specific issue that depends on the hypothesis being examined. Future work will examine our framework’s implementation in different contexts.

Our previous work developed a testing framework that detects whether the spatial location of key brain network regions and distributions of topological properties differ by phenotype (continuous and discrete) after controlling for confounding variables in single-task static networks. This work extends this framework to the multitask and multisession context by allowing it to handle multiple networks per individual while also displaying the utility of distance metrics using entire connection matrices (our previous work showed comparisons of nodal degree vectors). More generally, this framework allows relating distances between repeated observations of individual’s networks (e.g., Jaccard, KS distance) to their covariates of interest. Our proposed 3M_BANTOR method is computationally feasible and generally interpretable. We believe this extends an already convenient tool in the neuroscience toolbox to a more general class of problems.

## SUPPORTING INFORMATION

Supporting Information for this article is available at https://doi.org/10.1162/netn_a_00274. Simulation and HCP code is available at https://github.com/applebrownbetty/braindist_regression (Tomlinson et al., 2022). HCP data is publicly available for download.

## AUTHOR CONTRIBUTIONS

Chal E. Tomlinson: Conceptualization; Data curation; Formal analysis; Investigation; Methodology; Project administration; Resources; Supervision; Validation; Visualization; Writing – original draft; Writing – review & editing. Paul J. Laurienti: Data curation; Resources; Writing – original draft; Writing – review & editing. Robert G. Lyday: Data curation; Writing – original draft; Writing – review & editing. Sean L. Simpson: Conceptualization; Data curation; Formal analysis; Funding acquisition; Investigation; Methodology; Project administration; Resources; Supervision; Validation; Visualization; Writing – original draft; Writing – review & editing.

## FUNDING INFORMATION

Sean L. Simpson, National Institute of Biomedical Imaging and Bioengineering (https://dx.doi.org/10.13039/100000070), Award ID: R01EB024559. Sean L. Simpson, Wake Forest Clinical and Translational Science Institute, Wake Forest School of Medicine (https://dx.doi.org/10.13039/100019340), Award ID: UL1TR001420.

## TECHNICAL TERMS

Permutation testing framework: A method to test statistical significance utilizing permutation (switching labels).
Degree distribution: The probability distribution of the degree of nodes across a network.
Symmetric matrix: A matrix which entry *a*_*ij*_ = *a*_*ji*_ for all *i* and *j*. In the connectivity matrix space, this lets us know we are considering an undirected network.
Mixed model: Statistical model containing both fixed (population-level) and random (individual-level) effects used to model multivariate data.
Fixed effects: Variables whose effects are constant across individuals.
Random effect: Variable whose effect varies across individuals.
Empirical distribution function: Estimate of the cumulative distribution function using the data.
Logarithmic transformation: A fancy way of saying we took the log.
Power: Probability of correctly rejecting the null hypothesis.
Fair coin: A coin which has a 50% chance of landing on either side.
Beta distribution: A family of continuous probability distributions defined on the closed interval [0, 1].
Type I error rate: probability of incorrectly rejecting the null hypothesis.

## Supporting Information

https://github.com/applebrownbetty/braindist_regression
