# Supplementary material for: 3M_BANTOR: A regression framework for multitask and multisession brain network distance metrics
Source: Netw Neurosci. 2023 Jan 1;7(1):1–21. doi: 10.1162/netn_a_00274 (PMC10270667; doi:10.1162/netn_a_00274)
Supplement: Supplementary file 1 [file netn-7-1-1-s001.pdf]

Tomlinson, C. E., Laurienti, P. J., Lyday, R. G. & Simpson, S. L. (2023). Supporting information for "3M_BANTOR: A regression framework for Multitask and Multisession brain network distance metrics." *Network Neuroscience*, *7*(1), 1–21. https://doi.org/10.1162/netn_a_00274

## Title

3M_BANTOR: A Regression Framework for Multitask and Multisession Brain Network Distance Metrics

Supplemental

## Authors

Chal E. Tomlinson[1], Paul J. Laurienti[2,3], Robert G. Lyday[2,3], Sean L. Simpson[2,4,*]

[1] Department of Biostatistics, University of North Carolina at Chapel Hill, Chapel Hill, NC, USA

[2] Laboratory for Complex Brain Networks, Wake Forest School of Medicine, Winston-Salem, NC, USA.

[3] Department of Radiology, Wake Forest School of Medicine, Winston-Salem, NC, USA.

[4] Department of Biostatistics and Data Science, Wake Forest School of Medicine, Winston-Salem, NC, USA.

* **Corresponding Author**. Department of Biostatistics and Data Science, Wake Forest School of Medicine,

Winston-Salem, NC 27157, USA. Email: slsimpso@wakehealth.edu

Tomlinson, C. E., Laurienti, P. J., Lyday, R. G. & Simpson, S. L. (2022). Supporting information for "3M_BANTOR: A regression framework for Multitask and Multisession brain network distance metrics." *Network Neuroscience*. Advance publication. https://doi.org/10.1162/netn_a_00274

## 4.3. Results

| | Resting State | | | | | | | |
|---|---|---|---|---|---|---|---|---|
| | KS | | Jaccard | | Euclidean | | LERM | |
| | Est. | Std. Err. | Est. | Std. Err. | Est. | Std. Err. | Est. | Std. Err. |
| FluidIntl | -8.40E-05 | 1.21E-04 | 9.98E-05 | 2.42E-04 | 1.22E-02 | 1.47E-02 | -3.26E-03 | 8.39E-03 |
| Age | -1.59E-04 | 2.01E-04 | -1.92E-04 | 4.04E-04 | 2.02E-03 | 2.46E-02 | -1.04E-02 | 1.40E-02 |
| BMI | 1.26E-06 | 9.86E-05 | -3.91E-05 | 1.98E-04 | 9.86E-03 | 1.20E-02 | 5.38E-03 | 6.86E-03 |
| Education | -2.36E-04 | 3.34E-04 | -4.64E-04 | 6.69E-04 | -3.25E-02 | 4.08E-02 | -2.67E-02 | 2.32E-02 |
| Gender | -2.73E-04 | 9.44E-04 | 2.48E-03 | 1.89E-03 | 1.32E-01 | 1.15E-01 | 9.71E-02 | 6.57E-02 |
| Handedness | -1.50E-05 | 1.27E-05 | 4.65E-05 | 2.55E-05 | -5.27E-04 | 1.56E-03 | 9.35E-04 | 8.86E-04 |
| Income | -1.57E-04 | 2.36E-04 | -1.77E-04 | 4.73E-04 | -9.16E-03 | 2.88E-02 | -1.18E-02 | 1.64E-02 |
| Race | -2.58E-04 | 1.55E-03 | 5.47E-03 | 3.10E-03 | 5.63E-01 | 1.89E-01 | 2.76E-01 | 1.07E-01 |
| SmokeStatus | 2.43E-04 | 2.18E-03 | -3.48E-03 | 4.36E-03 | -1.24E-01 | 2.66E-01 | -1.61E-01 | 1.52E-01 |

*Table S1: Parameters and Standard Errors for HCP resting state brain scans when modeled with 3M_BANTOR and connectivity matrices.*

| | Working Memory (Phase LR) | | | | | | | |
|---|---|---|---|---|---|---|---|---|
| | KS | | Jaccard | | Euclidean | | LERM | |
| | Est. | Std. Err. | Est. | Std. Err. | Est. | Std. Err. | Est. | Std. Err. |
| FluidIntl | 2.05E-05 | 1.17E-04 | 2.25E-04 | 2.39E-04 | 2.07E-02 | 1.59E-02 | 1.79E-02 | 7.17E-03 |
| Age | -2.65E-04 | 1.95E-04 | -6.55E-04 | 3.99E-04 | -4.15E-02 | 2.65E-02 | 4.15E-03 | 1.20E-02 |
| BMI | -6.72E-05 | 9.56E-05 | 2.95E-04 | 1.95E-04 | 1.74E-02 | 1.30E-02 | 5.76E-03 | 5.86E-03 |
| Education | 3.12E-04 | 3.23E-04 | -1.27E-04 | 6.61E-04 | 1.27E-02 | 4.39E-02 | -7.55E-03 | 1.98E-02 |
| Gender | 1.58E-03 | 9.15E-04 | 1.28E-03 | 1.87E-03 | 1.16E-01 | 1.24E-01 | 4.35E-02 | 5.61E-02 |
| Handedness | -2.27E-05 | 1.23E-05 | -5.95E-06 | 2.52E-05 | -1.12E-03 | 1.68E-03 | 3.74E-05 | 7.57E-04 |
| Income | -3.19E-05 | 2.29E-04 | -1.39E-04 | 4.67E-04 | 1.61E-02 | 3.11E-02 | 1.13E-02 | 1.40E-02 |
| Race | -3.92E-04 | 1.50E-03 | 3.83E-03 | 3.06E-03 | 2.07E-01 | 2.03E-01 | 4.35E-02 | 9.19E-02 |
| SmokeStatus | 8.15E-06 | 2.11E-03 | -1.64E-03 | 4.32E-03 | -2.04E-01 | 2.87E-01 | -6.02E-02 | 1.29E-01 |

*Table S2: Parameters and Standard Errors for HCP working memory (phase LR) brain scans when modeled with 3M_BANTOR and connectivity matrices.*

Tomlinson, C. E., Laurienti, P. J., Lyday, R. G. & Simpson, S. L. (2022). Supporting information for “3M_BANTOR: A regression framework for Multitask and Multisession brain network distance metrics.” *Network Neuroscience*. Advance publication. https://doi.org/10.1162/netn_a_00274

| | Working Memory (Phase RL) | | | | | | | |
|---|---|---|---|---|---|---|---|---|
| | KS | | Jaccard | | Euclidean | | LERM | |
| | Est. | Std. Err. | Est. | Std. Err. | Est. | Std. Err. | Est. | Std. Err. |
| FluidIntl | -3.79E-05 | 1.17E-04 | 4.33E-05 | 2.20E-04 | 1.54E-02 | 1.27E-02 | 6.51E-03 | 6.66E-03 |
| Age | -6.22E-05 | 1.95E-04 | -3.50E-04 | 3.67E-04 | -4.12E-04 | 2.12E-02 | -1.48E-02 | 1.11E-02 |
| BMI | 5.71E-05 | 9.55E-05 | 3.98E-04 | 1.80E-04 | 1.87E-02 | 1.04E-02 | 1.25E-02 | 5.45E-03 |
| Education | -1.44E-04 | 3.23E-04 | -4.03E-04 | 6.08E-04 | -3.75E-02 | 3.51E-02 | -2.59E-03 | 1.84E-02 |
| Gender | -1.53E-04 | 9.15E-04 | 1.35E-04 | 1.72E-03 | -8.90E-02 | 9.93E-02 | 4.16E-02 | 5.22E-02 |
| Handedness | 2.42E-07 | 1.23E-05 | -1.31E-05 | 2.32E-05 | -2.75E-03 | 1.34E-03 | 1.55E-04 | 7.03E-04 |
| Income | -1.66E-04 | 2.29E-04 | 1.55E-04 | 4.30E-04 | -9.21E-03 | 2.48E-02 | 1.95E-03 | 1.30E-02 |
| Race | -1.54E-04 | 1.50E-03 | 3.38E-04 | 2.82E-03 | 4.14E-01 | 1.63E-01 | 2.08E-01 | 8.54E-02 |
| SmokeStatus | -1.33E-03 | 2.11E-03 | 4.48E-03 | 3.97E-03 | 4.73E-02 | 2.29E-01 | -2.12E-01 | 1.20E-01 |

*Table S3: Parameters and Standard Errors for HCP working memory (phase RL) brain scans when modeled with 3M_BANTOR and connectivity matrices.*

## 4.4. Results – Nodal Degree Vectors

Nodal degree vectors (used for the KS and Euclidean distances) were created by summing across rows of the connectivity matrices. Key nodes of interest (binary degree vectors used for the Jaccard distance) based on node degree were identified, selecting the top 5% or the top 20% highest degree (hub) nodes and mapping those to 1 while mapping all remaining nodes to 0. KS statistic and Euclidean distance were calculated for each pair of individuals using their nodal degree vectors. The Jaccard distance was calculated for each pair of individuals using their binary degree vectors.

Distance covariates for each pair of individuals were calculated. A continuous variable’s distance (Age, for instance) was calculated as $\left|Age_i - Age_j\right|$ for the pair of individuals $i$ and $j$. A binary or categorical variable’s distance (Education, for instance) was calculated as $\mathbb{1}\{Edu_i \neq Edu_j\}$ for the pair of individuals $i$ and $j$.

Tomlinson, C. E., Laurienti, P. J., Lyday, R. G. & Simpson, S. L. (2022). Supporting information for "3M_BANTOR: A regression framework for Multitask and Multisession brain network distance metrics." *Network Neuroscience*. Advance publication. https://doi.org/10.1162/netn_a_00274

We evaluated differences between networks with our proposed 3M_BANTOR approach. Resting state fMRI were compared between all individuals for both sessions (1 and 2) and phases (LR and RL). Working memory block design was different between the RL and LR phases, so we did not compare working memory connection matrices between phases. Thus, covariates were estimated for resting state (combining both phases), working memory (phase LR) and working memory (phase RL). Parameter and standard error estimates can be found in Tables S5, S6 and S7. Each parameter estimate represented the average amount the given brain distance metric (KS, Jaccard, etc.) changed based on a one-unit difference in the respective covariate, after controlling for other covariates. A complete list of p-values for both resting state and working memory can be seen in Table S4. Given the high degree of dependence between these results, and the illustrative and exploratory nature of our analysis, there have been no adjustments for multiple comparisons.

| | Resting State – Nodal Degree | | | |
|---|---|---|---|---|
| | KS | JAC_05 | JAC_20 | EUC |
| FluidIntl | 3.46E-01 | 2.85E-01 | 4.14E-01 | 8.11E-01 |
| Age | 4.66E-01 | 2.65E-01 | 3.57E-01 | 3.78E-01 |
| BMI | 8.15E-01 | 6.13E-01 | 7.38E-01 | 8.59E-01 |
| Education | 5.08E-01 | 3.11E-01 | 5.08E-01 | 1.97E-01 |
| Gender | 8.32E-01 | 9.71E-02 | 1.93E-01 | 8.04E-01 |
| Handedness | 4.08E-01 | 9.56E-01 | 8.90E-01 | 5.82E-01 |
| Income | 8.35E-01 | 3.13E-01 | 2.86E-01 | 3.51E-01 |
| Race | 7.81E-01 | 6.01E-01 | 6.88E-01 | 7.07E-01 |
| SmokeStatus | 7.65E-01 | 7.03E-01 | 4.87E-01 | 5.32E-01 |

| | Working Memory (Phase LR) – Nodal Degree | | | | Working Memory (Phase RL) | | | |
|---|---|---|---|---|---|---|---|---|
| | KS | JAC_05 | JAC_20 | EUC | KS | JAC_05 | JAC_20 | EUC |
| FluidIntl | 9.79E-01 | 6.80E-01 | 4.10E-01 | 5.15E-01 | 7.52E-01 | 5.05E-01 | 3.20E-02 | 7.97E-01 |
| Age | 1.21E-01 | 8.48E-02 | 1.41E-01 | 4.80E-02 | 7.26E-01 | 3.61E-01 | 1.93E-01 | 4.84E-01 |
| BMI | 7.13E-01 | 9.16E-01 | 4.90E-01 | 5.07E-01 | 4.14E-01 | 3.00E-01 | 1.42E-01 | 2.78E-01 |
| Education | 6.16E-01 | 1.26E-01 | 8.45E-01 | 6.23E-01 | 7.06E-01 | 4.08E-01 | 4.48E-01 | 6.40E-01 |
| Gender | 7.31E-02 | 6.88E-03 | 8.75E-02 | 4.20E-03 | 9.60E-01 | 5.24E-01 | 6.57E-01 | 5.92E-01 |

Tomlinson, C. E., Laurienti, P. J., Lyday, R. G. & Simpson, S. L. (2022). Supporting information for "3M_BANTOR: A regression framework for Multitask and Multisession brain network distance metrics." *Network Neuroscience*. Advance publication. https://doi.org/10.1162/netn_a_00274

| | | | | | | | | |
|---|---|---|---|---|---|---|---|---|
| Handedness | 8.34E-02 | 2.43E-01 | 3.23E-01 | 4.29E-01 | 9.39E-01 | 7.93E-01 | 4.21E-01 | 6.87E-01 |
| Income | 7.05E-01 | 4.60E-01 | 3.49E-01 | 4.24E-01 | 2.88E-01 | 5.83E-01 | 9.99E-01 | 6.27E-01 |
| Race | 5.73E-01 | 3.10E-01 | 9.48E-01 | 9.36E-01 | 7.07E-01 | 4.57E-02 | 6.50E-02 | 5.90E-01 |
| SmokeStatus | 5.54E-01 | 3.18E-01 | 9.85E-01 | 8.94E-01 | 3.05E-01 | 3.20E-01 | 4.83E-01 | 7.40E-01 |

Legend 0 0.05 1

*Table S1: P-values for HCP resting state, working memory (phase LR) and working memory (phase RL) brain scans when modeled with 3M_BANTOR and nodal degree vectors. Parameter estimates and standard errors can be found in tables S5, S6, and S7.*

Fluid Intelligence had a statistically significant relationship for Jaccard Distance (top 20%) during Working Memory (Phase RL), but did not have a statistically significant relationship with any other distance metric (KS, Jaccard Distance (top 5%), Euclidean) for Resting State or Working Memory fMRI when distances were calculated using nodal degree vectors.

| | Resting State - Nodal Degree | | | | | | | |
|---|---|---|---|---|---|---|---|---|
| | KS | | Jaccard Top 5% | | Jaccard Top 20% | | Euclidean | |
| | Est. | Std. Err. | Est. | Std. Err. | Est. | Std. Err. | Est. | Std. Err. |
| FluidIntl | -5.98E-04 | 6.34E-04 | 4.40E-04 | 4.11E-04 | 3.13E-04 | 3.82E-04 | -3.00E-02 | 1.25E-01 |
| Age | -7.73E-04 | 1.06E-03 | -7.67E-04 | 6.86E-04 | -5.89E-04 | 6.39E-04 | -1.84E-01 | 2.09E-01 |
| BMI | 1.22E-04 | 5.18E-04 | -1.70E-04 | 3.36E-04 | 1.05E-04 | 3.13E-04 | -1.82E-02 | 1.02E-01 |
| Education | -1.16E-03 | 1.75E-03 | -1.15E-03 | 1.14E-03 | -7.02E-04 | 1.06E-03 | -4.48E-01 | 3.46E-01 |
| Gender | -1.06E-03 | 4.96E-03 | 5.35E-03 | 3.22E-03 | 3.91E-03 | 3.00E-03 | 2.44E-01 | 9.80E-01 |
| Handedness | -5.55E-05 | 6.69E-05 | -2.40E-06 | 4.34E-05 | -5.60E-06 | 4.04E-05 | -7.29E-03 | 1.32E-02 |
| Income | -2.58E-04 | 1.24E-03 | -8.13E-04 | 8.04E-04 | -8.00E-04 | 7.48E-04 | -2.29E-01 | 2.45E-01 |
| Race | -2.26E-03 | 8.12E-03 | 2.76E-03 | 5.27E-03 | 1.97E-03 | 4.90E-03 | 6.03E-01 | 1.60E+00 |
| SmokeStatus | 3.43E-03 | 1.14E-02 | -2.83E-03 | 7.42E-03 | 4.81E-03 | 6.91E-03 | 1.41E+00 | 2.26E+00 |

*Table S5: Parameters and Standard Errors for HCP resting state brain scans when modeled with 3M_BANTOR and nodal degree vectors.*

Tomlinson, C. E., Laurienti, P. J., Lyday, R. G. & Simpson, S. L. (2022). Supporting information for “3M_BANTOR: A regression framework for Multitask and Multisession brain network distance metrics.” *Network Neuroscience*. Advance publication. https://doi.org/10.1162/netn_a_00274

| | Working Memory (Phase LR) - Nodal Degree | | | | | | | |
|---|---|---|---|---|---|---|---|---|
| | KS | | Jaccard Top 5% | | Jaccard Top 20% | | Euclidean | |
| | Est. | Std. Err. | Est. | Std. Err. | Est. | Std. Err. | Est. | Std. Err. |
| FluidIntl | -1.89E-05 | 7.26E-04 | 2.18E-04 | 5.28E-04 | 4.01E-04 | 4.86E-04 | 8.31E-02 | 1.28E-01 |
| Age | -1.88E-03 | 1.21E-03 | -1.53E-03 | 8.82E-04 | -1.20E-03 | 8.13E-04 | -4.22E-01 | 2.13E-01 |
| BMI | -2.19E-04 | 5.94E-04 | 4.56E-05 | 4.32E-04 | 2.75E-04 | 3.98E-04 | -6.93E-02 | 1.04E-01 |
| Education | 1.01E-03 | 2.01E-03 | -2.25E-03 | 1.46E-03 | 2.63E-04 | 1.35E-03 | 1.74E-01 | 3.53E-01 |
| Gender | 1.02E-02 | 5.68E-03 | 1.13E-02 | 4.14E-03 | 6.53E-03 | 3.81E-03 | 2.87E+00 | 1.00E+00 |
| Handedness | -1.33E-04 | 7.67E-05 | 6.52E-05 | 5.58E-05 | 5.09E-05 | 5.14E-05 | -1.07E-02 | 1.35E-02 |
| Income | -5.37E-04 | 1.42E-03 | 7.65E-04 | 1.03E-03 | 8.93E-04 | 9.52E-04 | 2.00E-01 | 2.50E-01 |
| Race | -5.25E-03 | 9.30E-03 | -6.88E-03 | 6.77E-03 | -4.07E-04 | 6.24E-03 | 1.31E-01 | 1.64E+00 |
| SmokeStatus | 7.76E-03 | 1.31E-02 | -9.54E-03 | 9.54E-03 | 1.62E-04 | 8.79E-03 | -3.06E-01 | 2.31E+00 |

*Table S6: Parameters and Standard Errors for HCP working memory (phase LR) brain scans when modeled with 3M_BANTOR and nodal degree vectors.*

| | Working Memory (Phase RL) - Nodal Degree | | | | | | | |
|---|---|---|---|---|---|---|---|---|
| | KS | | Jaccard Top 5% | | Jaccard Top 20% | | Euclidean | |
| | Est. | Std. Err. | Est. | Std. Err. | Est. | Std. Err. | Est. | Std. Err. |
| FluidIntl | -2.17E-04 | 6.88E-04 | 3.36E-04 | 5.05E-04 | 1.03E-03 | 4.77E-04 | 3.91E-02 | 1.52E-01 |
| Age | -4.03E-04 | 1.15E-03 | -7.71E-04 | 8.43E-04 | -1.04E-03 | 7.97E-04 | -1.78E-01 | 2.54E-01 |
| BMI | 4.61E-04 | 5.63E-04 | 4.28E-04 | 4.13E-04 | 5.75E-04 | 3.90E-04 | 1.35E-01 | 1.24E-01 |
| Education | -7.18E-04 | 1.91E-03 | -1.16E-03 | 1.40E-03 | -1.00E-03 | 1.32E-03 | -1.97E-01 | 4.21E-01 |
| Gender | -2.68E-04 | 5.39E-03 | -2.52E-03 | 3.95E-03 | -1.66E-03 | 3.74E-03 | -6.38E-01 | 1.19E+00 |
| Handedness | 5.60E-06 | 7.27E-05 | -1.40E-05 | 5.33E-05 | -4.06E-05 | 5.04E-05 | 6.48E-03 | 1.61E-02 |
| Income | -1.43E-03 | 1.35E-03 | -5.43E-04 | 9.87E-04 | 1.35E-06 | 9.34E-04 | -1.45E-01 | 2.98E-01 |
| Race | -3.32E-03 | 8.82E-03 | 1.29E-02 | 6.47E-03 | 1.13E-02 | 6.12E-03 | 1.05E+00 | 1.95E+00 |
| SmokeStatus | -1.28E-02 | 1.24E-02 | -9.07E-03 | 9.12E-03 | -6.05E-03 | 8.62E-03 | -9.14E-01 | 2.75E+00 |

*Table S7: Parameters and Standard Errors for HCP working memory (phase RL) brain scans when modeled with 3M_BANTOR and nodal degree vectors.*
